# Sirolimus versus cyclosporine A in patients with primary acquired pure red cell aplasia: a prospective cohort study

**DOI:** 10.1038/s41408-023-00845-3

**Published:** 2023-05-10

**Authors:** Yuan Yang, Zengwei Tang, Yuzhou Huang, Qinglin Hu, Shuqing Wang, Jiang Ji, Yali Du, Chen Yang, Miao Chen, Shimin Hu, Bing Han

**Affiliations:** 1 Department of Hematology, Peking Union Medical College Hospital, Chinese Academy of Medical Sciences and Peking Union Medical College, Beijing, 100730 China; 2 Department of Hepatobiliary and Pancreatic Surgery, the First Affiliated Hospital, School of Medicine, Zhejiang University, Hangzhou, 310003 Zhejiang China; 3 Department of Gastroenterology, Sun Yat-sen Memorial Hospital, Sun Yat-sen University, Guangzhou, China; 4 Department of Hematopathology, The University of Texas MD Anderson Cancer Center, Houston, TX USA

**Keywords:** Phase IV trials, Molecularly targeted therapy

Dear Editor

Pure red cell aplasia (PRCA) is characterized by normocytic normochromic anemia, reticulocytopenia, and absence of erythroid precursors in the bone marrow [1]. It can be classified into congenital or acquired PRCA (aPRCA) according to the pathogenesis and etiologies. Acquired PRCA can be further stratified into primary and secondary ones [2]. aPRCA can be secondary to a variety of diseases including thymoma, large granular lymphocytic leukemia, parvovirus B19 infection, drug uses, hematological or immune diseases, solid tumors, etc [2, 3]. Primary aPRCA refers to those with no detectable underlying diseases [2, 4].

Currently, the first-line agents used for the treatment of aPRCA include cyclosporine A (CsA) and corticosteroid. However, corticosteroid alone does not have durable efficacy and may result in severe side effects [3]. CsA, as a first-line treatment, is widely used to treat aPRCA patients with a response rate of ~65–85% [2–4]. However, about 20–30% of patients treated with CsA relapse later when CsA is tapered or withdrawn [2]. In addition, some patients cannot tolerate CsA either due to severe side effects, advanced age, or renal dysfunction, and have to rely on blood transfusion.

Sirolimus (rapamycin), a lipophilic macrolide antibiotic synthesized by *streptomyces hygroscopicus*, has strong immunosuppressive and anti-cell proliferation activities and has been used for antitumor therapy and post-transplantation immunosuppression [5, 6]. We recently demonstrated that sirolimus is an effective treatment for patients with refractory /relapsed/intolerant aPRCA [7]. Even patients who are refractory to CsA may respond to sirolimus, indicating that sirolimus may have a different mechanism of action from that of CsA. So far, no study has been conducted on the use of sirolimus to treat newly diagnosed aPRCA.

In this prospective cohort study (NCT04470804), we evaluated the safety and efficacy of sirolimus versus CsA as the frontline therapy for patients diagnosed with primary aPRCA. A total of 68 patients were screened for eligibility. Among these, 11 patients were excluded for not meeting the inclusion criteria. The remaining 57 patients were ultimately enrolled: 27 were assigned to the sirolimus group and the other 30 were assigned to the CsA group. One patient in the sirolimus group was lost after 3-month treatment and was excluded from the final analysis. The remaining 56 patients all finished the designed treatment and were followed up for at least 1 year (Fig. 1A). The details of patient selection, therapy regimens, laboratory tests, evaluation of response and monitoring of toxicity, sample size calculation and statistical analyses in this clinical trial were included in Supplementary Methods.

**Fig. 1:**
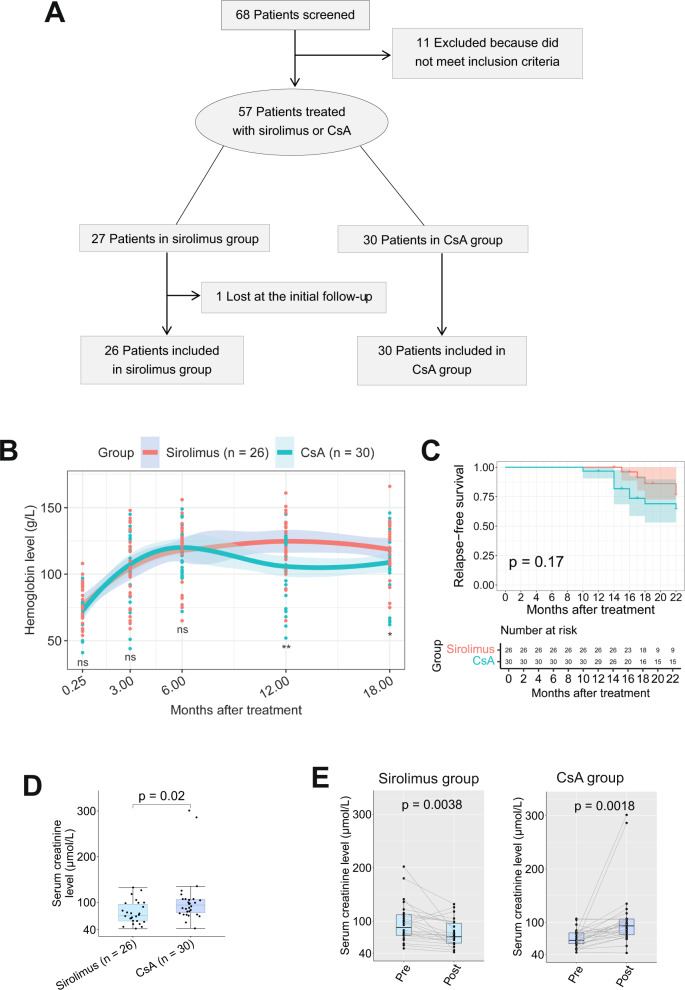
Patient screening and assignment, and comparison of hemoglobin level, relapse-free survival or serum creatinine level in patients with sirolimus or CsA treatment. **A** Trial profile. A total of 68 patients were screened for the eligibility. Among those, 11 patients were excluded for not meeting the inclusion criteria, and the remaining 57 patients were ultimately enrolled. Of those, 30 patients received CsA therapy, and the others received sirolimus therapy. Except one patient in sirolimus group lost at the initial follow-up, the remaining patients had completed the designed treatment. **B** Hemoglobin change after sirolimus or CsA treatment. Dot plot shows the change of hemoglobin level over time in patients treated with sirolimus (*n* = 26) or CsA (*n* = 30). ns, *P* > 0.05, *P < 0.05, **P < 0.01, using two-tailed unpaired student’s *t*-test. The trendline denotes the median value of hemoglobin and the shade area around the trendline denotes the 95% confident interval of the corresponding median value in each group. **C** Relapse-free survival of patients treated with sirolimus (*n* = 26) or CsA (*n* = 30). *P* was based on log-rank test. **D** Serum creatinine level in patients post sirolimus and CsA treatment. Boxplot shows serum creatinine level in patients post treatment with sirolimus (*n* = 26) or CsA (*n* = 30). *P* was based on two-tailed unpaired student’s *t*-test. **E** Serum creatinine level in patients pre/post sirolimus or CsA treatment, respectively. Boxplot shows serum creatinine level in patients pre/post-treatment with sirolimus (*n* = 26) or CsA (*n* = 30). *P* was based on two-tailed paired student’s *t*-test.

In the sirolimus group (*n* = 26), there were 14 males and 12 females with a median age of 66 years (range, 37–88). The mean hemoglobin (HGB), white blood cell count, and platelet count were 74.5 ± 12.5 g/L, (6.4 ± 2.4) × 10^9^/L, (260.6 ± 71.8) × 10^9^/L, respectively. The frequency of STAT3 mutation was 15.3% (4/26). In the CsA group (*n* = 30), there were 19 males and 11 females with a median age of 65.5 years (range, 18–77). The mean HGB, white blood cell count, and platelet count were 71.1 ± 14.0 g/L, (6.0 ± 1.8) × 10^9^/L, (273.5 ± 112.3) × 10^9^/L, respectively, and the prevalence of STAT3 mutation was 6.6% (2/30). The baseline clinical features between the two groups were similar except that the Cr level was significantly higher in the sirolimus group (*P* = 0.002, Table 1) according to the patient selection criteria in Supplementary Methods. The median follow-up time was 18 months (range, 12–22) in the sirolimus group and 20 months (range, 14–22) in the CsA group (*P* = 0.518). The median time to response was 3 months (range, 1–12) in the sirolimus group and 2.5 months (range, 1–9) in the CsA group (*P* = 0.784).

**Table 1:** Baseline clinical characteristics of patients.

Variable	All patients (*n* = 56)	Sirolimus group (*n* = 26)	CsA group (*n* = 30)	*P*
Age, median (range)	65 (18–88)	66 (37–88)	65.5 (18–77)	0.090
Male, *n* (%)	33 (58.9)	14 (53.8)	19 (63.3)	0.987
Ret# (×10^9^/L)	12.4 (1–25.4)	12.0 (4.3–22.5)	15.2 (1–25.4)	0.441
HGB, g/L	72.8 ± 13.6	74.5 ± 12.5	71.1 ± 14.0	0.339
WBC (×10^9^/L)	6.2 ± 2.0	6.4 ± 2.4	6.0 ± 1.8	0.390
PLT (×10^9^/L)	262.9 ± 99.9	260.6 ± 71.8	273.5 ± 112.3	0.617
Cr, μmol/L	79.2 ± 27.3	94.1 ± 38.2	69.8 ± 16.1	0.002
LDH (U/L)	229.2 ± 53.7	220.7 ± 51.1	224.9 ± 53.9	0.765
Ferroprotein, ng/ml	1121 ± 160.2	1638 ± 416.1	1084 ± 243.4	0.275
STAT3 mutation, *n* (%)	6 (10.7%)	4 (15.3%)	2 (6.6%)	0.658
Response time, months, median (range)	2.5 (1–12)	3 (1–12)	2.5 (1–9)	0.784
Follow-up period, months, median (range)	18 (12–22)	18 (14–22)	20 (12–22)	0.518

*CsA* Cyclosporine A, *Ret* reticulocyte, *HGB* Hemoglobin, *WBC* white blood cell, *PLT* platelet, *Cr* creatinine, *LDH* lactate dehydrogenase.

The overall response rate (ORR) at 3, 6, and 12 months and the end of the follow-up were 42.3%, 73.1%, 80.8%, 73.1% in the sirolimus group and 56.7%, 76.7%, 60.0%, 56.7% in the CsA group, respectively. The complete response rates (CRR) at these time-points were 30.8%, 73.1%, 73.1%, and 69.2% in the sirolimus group and 46.7%, 60.0%, 43.3%, and 40.0% in the CsA group, respectively. The ORR and CRR at 3 and 6 months were similar in the two groups, whereas the ORR and CRR at 12 months were significantly higher in the sirolimus group (80.8% *vs* 60.0%, *P* = 0.039; 73.1% *vs*. 43.3%, *P* = 0.035, respectively; Supplementary Table S1). Furthermore, the HGB level at 12-month was significantly higher in the sirolimus group (124.7 ± 22.2 *vs*. 105.8 ± 23.1 g/L, *P* = 0.0029; Fig. 1B).

Four (15.3%) patients in the sirolimus group relapsed at a median of 17.5 months (range, 15–22). Among them, three patients relapsed during the tapering period (0.5 mg/d): two patients responded again after the dose was increased, and the other patient did not response and remained transfusion dependent. One patient who had response to sirolimus relapsed even with full dose of sirolimus and he was dependent on transfusion afterwards. Nine (30.0%) patients in the CsA group relapsed at a median of 14 months (range, 10–22). Among them, seven patients relapsed during tapering period (75 mg/d), either from regular tapering or drug intolerance: three patients switched to sirolimus and achieved CR after 2–4 months, and the other four patients did not respond again after the CsA dose was increased and became dependent on transfusion. The remaining two patients relapsed even with a full dose of CsA, then switched to sirolimus, and achieved CR at 3 months.

Patients in the sirolimus group had a lower relapse rate than those in the CsA group (15.3% vs. 30.0%, *P* = 0.044). Additionally, patients in the sirolimus group showed a trend toward a longer relapse-free survival than patients in the CsA group (*P* = 0.17, Fig. 1C). No clonal evolution, death or serious adverse events was observed in either group at the end of follow-up.

Drug-related adverse events were not significantly different between the two groups (Supplementary Table S2). The baseline Cr level was significantly higher in sirolimus group compared with CsA group (Table 1). Nevertheless, the Cr level was significantly lower in sirolimus group relative to CsA group after treatment (Fig. 1D), indicating that sirolimus can improve the renal function of aPRCA patients. Additionally, the Cr level was significantly improved in the sirolimus group (96.5 ± 37.1 *vs* 76.8 ± 24.8 μmol/L, *P* = 0.0038) whereas it was significantly deteriorated in the CsA group (69.8 ± 16.1 *vs*. 106.2 ± 55.2 μmol/L, *P* = 0.0018) at the end of follow-up (Fig. 1E).

The serum level of TNF-α and EPO were significantly decreased in sirolimus group whereas not markedly changed in CsA group at 6 months post-treatment; CD4^+^/CD8^+^ T cell ratio and levels of IL-6/810 were not significantly changed at 6 months post-treatment in both sirolimus and CsA group (Supplementary Table S3). Consistent with our finding, Feng et al. [8]. has also found that sirolimus can effectively decrease TNF-α in preclinical mouse models with immune-mediated bone marrow failure. Additionally, we observed a higher HGB level in sirolimus than CsA group at 12-month (Fig. 1B). This may partly explain the lower EPO level after sirolimus, considering that EPO is mainly produced by kidneys in response to anemic hypoxia [9, 10]. Whether the decrease of TNF-α and EPO levels in aPRCA patients correlated with favorable outcomes in the sirolimus group is unclear. The findings in MDS that serum levels of EPO above 200 pg/ml and high serum TNF-α concentrations predict shortened survival in high-risk MDS patients may provide some hints [11]. These data, in combination with our previous data showing that the Treg levels in CsA-refractory patients increased significantly after effective salvage therapy with sirolimus [12], indicate that sirolimus may act differently, and it may rescue some patients resistant to CsA.

There are also some limitations in our study. First, this is a single-center study with limited number of patients, and some potential factors that may affect the outcome cannot be estimated. Second, patients with baseline renal dysfunction had been assigned to the sirolimus group, which may have caused imbalance between the two groups to some extent. Third, due to practical concerns (the toxicity, low trial enrollment, and difficulty to give medications caused by the COVID-19 pandemic) and clinical considerations (the treatment efficacy was better in sirolimus group than CsA group, thus we had reached our primary endpoint, and considering that the potential impact of COVID-19 infection on patients such as the anemia [13, 14] in patients post COVID-19 infection or COVID-19 mRNA vaccination [15]), we terminated our study following the interim analysis of the clinical result of 57 participants. Finally, the follow-up time was relatively short for assessing long-term effectiveness, relapse and survival.

In conclusion, our results support that sirolimus could be used to treat patients with newly diagnosed primary aPRCA, especially those with renal dysfunction.

## Supplementary information

Supplementary Methods

Table S1

Table S2

Table S3

## Data Availability

All data are available in the main text. All detailed metadata are available upon reasonable request to corresponding author H.B.
