# Supplementary material for: Sirolimus versus cyclosporine A in patients with primary acquired pure red cell aplasia: a prospective cohort study
Source: Blood Cancer J. 2023 May 10;13(1):74. doi: 10.1038/s41408-023-00845-3 (PMC10169841; doi:10.1038/s41408-023-00845-3)
Supplement: Supplementary file 1 — Supplementary Methods [file 41408_2023_845_MOESM1_ESM.docx]

**Sirolimus versus cyclosporine A in patients with primary acquired pure red cell aplasia: a prospective cohort study**

Yuan Yang, Zengwei Tang, Yuzhou Huang, Qinglin Hu, Shuqing Wang, Jiang Ji, Yali Du, Chen Yang, Miao Chen, Shimin Hu, and Bing Han

**Methods**

**Patient selection**

Patients who were newly diagnosed with primary aPRCA at Peking Union Medical College Hospital (PUMCH) from July 2020 through April 2021 were prospectively enrolled according to the following criteria: (1) age >18 years old; (2) hemoglobin (Hb) <90 g/L before treatment; (3) no history of immunosuppression therapy prior to sirolimus or CsA treatment; (4) adequate hepatic functions with alanine transaminase (ALT)/aspartate transaminase (AST) levels within 3 times of the normal upper limit and total bilirubin levels within 2 times of the normal upper limit; (5) documented patient consent. The exclusion criteria were as following: (1) diagnosis of secondary aPRCA; (2) history of treatment with immunosuppression therapy before enrollment; (3) history of leukemia, stem cell transplantation, or treatment-related myelodysplastic syndromes (MDS); (4) creatinine/transaminase ≥ 3 normal upper limit; (5) complicated with active or uncontrolled infections or uncontrolled cardiovascular disease; (6) presence of concomitant malignancies such as thymoma, and T-cell large granular lymphocyte leukemia (LGL); (7) presence of other diseases that may cause anemia; and (8) pregnant and lactating women. The diagnosis of aPRCA was based on the criteria confirmed by bone marrow smear and biopsy as previously described [1].

After signing the consent forms, the patients were divided into either sirolimus group or CsA group based on the computer-generated random numbers. However, patients with renal insufficiency (the creatinine (Cr) level was up to 2 times of the normal upper limit) were assigned only to the sirolimus arm given our previous findings that sirolimus can reduce the serum level of Cr in aPRCA patients who were complicated with renal insufficiency post-CsA treatment [1,2]. The study design was approved by the Committee for the Ethical Review of Research at PUMCH. The study was registered at clinicaltrial.gov (<https://www.clinicaltrials.gov/ct2/show/NCT04470804?term=NCT04470804&draw=2&rank=1>).

**Therapy regimens**

Sirolimus was administered at a dose of 1-3 mg/d and the trough plasma concentration was maintained at 4-12 ng/ml. CsA was administered at 3-5 mg/(kg/d) and the trough plasma blood concentration was maintained at 100-200 ng/ml. Sirolimus or CsA treatment was continued for at least 6 months prior to the evaluation of treatment efficacy, and the treatments were administered continuously for at least 12 months once Hb reached at least partial remission after treatment and was gradually tapered afterwards. The patient may be transfused if the Hb level was lower than 60 g/L if possible or under the necessary conditions.

**Laboratory tests**

Patient data were recorded, including demographic characteristics, medical history, physical examination data, and laboratory test results including complete blood cell count, serum biochemistry such as liver and kidney functions and ferritin level, bone marrow smear and biopsy, chromosomal analysis, and myeloid malignancy gene mutation profile if possible. The patients in both groups were followed up monthly for at least 6 months after treatment was started and then every 3 months for at least one year for evaluation of response and side effects. Absolute CD4^+^ and CD8^+^ T cell counts, cytokines levels (IL-6, IL-8, IL-10, and TNF-α) and erythropoietin (EPO) levels were measured before and 6 months after treatment by flow cytometry or enzyme linked immunosorbent assay accordingly. Bone marrow smear, biopsy, and chromosome tests were repeated every 6 months, or if necessary, to exclude clonal evolution. Additional baseline workups included tests for hepatitis virus, cytomegalovirus, EBV, and parvovirus B19 as well as immune tests to exclude connective tissue diseases; CT/MRI scan of chest and abdomen, or even gastrointestinal endoscopy examination would be performed if necessary to rule out thymoma, lymphoma and other solid malignancies as we previously described [1,2]. All treatment-related side effects and laboratory data were obtained from the medical records in PUMCH.

**Evaluation of response and monitoring of toxicity**

Primary endpoint of this study was treatment response which was defined as following: complete response (CR) was defined as Hb >120 g/L in males or Hb >110 g/L in females and lasting for at least 2 months; partial response (PR) was defined as Hb levels that increased over 30 g/L compared with baseline levels and the achievement of a transfusion-independent status but not reaching the CR criteria [3]; no response (NR) was defined as not reaching the PR criteria; overall response (OR) included both complete and partial response; and relapse was defined as new dependence on transfusion.

Secondary endpoint of this study was safety that was assessed by analyzing the incidence and severity of adverse events, and was classified according to the National Cancer Institute Common Toxicity Criteria for Adverse Events version 5.0 [4].

**Sample size calculation and statistical analyses**

We assumed that the overall response in patients treated with sirolimus might be 1.5 times of that in those treated with CsA (based on experience with the treatment of chronic immune thrombocytopenia) [5,6]. A power calculation with PASS v.15.0. (NCSS, LLC, USA) indicated that 56 patients were required in each group to have a 95% chance of rejecting the hypothesis. After we recruited 57 patients, an interim analysis showed that patients in the sirolimus group already received significant therapy benefits compared with patients in the CsA group at 12 months.

Summary statistics for patient demographics and laboratory measurements were presented using the median, range, percentage, or means ± standard deviation. Intermediate analyses were performed to determine the statistical power of therapeutic differences between the sirolimus and CsA groups. Fisher’s exact test or the chi-square test and Student’s t test were used to calculate the significance of the differences between these two groups. Covariate effects on the response rate were evaluated using the univariable logistic regression with statistical inference presented using the corresponding standard errors. A *p* value <0.05 was considered statistically significant. All the statistical analyses were performed with SPSS v.22.0 (IBM Corp, Armonk, NY, USA) or R v.4.1.0 (R Foundation for Statistical Computing, Vienna, Austria).

**References**

1. Huang Y, Chen M, Yang C, Ruan J, Wang S, Han B. Sirolimus is effective for refractory/relapsed/intolerant acquired pure red cell aplasia: results of a prospective single-institutional trial. Leukemia*.* 2022;36(5):1351-1360.
2. Chen Z, Liu X, Chen M, Yang C, Han B. Successful sirolimus treatment of patients with pure red cell aplasia complicated with renal insufficiency. Ann Hematol*.* 2020;99(4):737-741.
3. Means RT, Jr. Pure red cell aplasia. Blood. 2016;128(21):2504-2509.
4. Freites-Martinez A, Santana N, Arias-Santiago S, Viera A. Using the Common Terminology Criteria for Adverse Events (CTCAE - Version 5.0) to Evaluate the Severity of Adverse Events of Anticancer Therapies. Actas Dermosifiliogr (Engl Ed). 2021;112(1):90-92.
5. Mousavi-Hasanzadeh M, Bagheri B, Mehrabi S, Eghbali A, Eghbali A. Sirolimus versus cyclosporine for the treatment of pediatric chronic immune thrombocytopenia: A randomized blinded trial. Int Immunopharmacol*.* 2020;88:106895.
6. Jasinski S, Weinblatt ME, Glasser CL. Sirolimus as an Effective Agent in the Treatment of Immune Thrombocytopenia (ITP) and Evans Syndrome (ES): A Single Institution's Experience. J Pediatr Hematol Oncol*.* 2017;39(6):420-424.
