# Supplementary material for: Sirolimus versus cyclosporine A in patients with primary acquired pure red cell aplasia: a prospective cohort study
Source: Blood Cancer J. 2023 May 10;13(1):74. doi: 10.1038/s41408-023-00845-3 (PMC10169841; doi:10.1038/s41408-023-00845-3)
Supplement: Supplementary file 2 — Table S1 [file 41408_2023_845_MOESM2_ESM.doc]

| **Table S1. Summary of response rate in patients with sirolimus or CsA** | | | | | | |
| --- | --- | --- | --- | --- | --- | --- |
|  | ORR (n, %) | | *P* | CRR (n, %) | | *P* |
|  | Sirolimus group | CsA group |  | Sirolimus group | CsA group |  |
| 3-month | 12 (42.3) | 17 (56.7) | 0.422 | 8 (30.8) | 14 (46.7) | 0.279 |
| 6-month | 19 (73.1) | 23 (76.7) | 0.768 | 19 (73.1) | 18 (60.0) | 0.399 |
| 12-month | 21 (80.8) | 18 (60.0) | 0.039 | 19 (73.1) | 13 (43.3) | 0.035 |
| End point | 19 (73.1) | 17 (56.7) | 0.088 | 18 (69.2) | 12 (40.0） | 0.052 |
| ORR, Overall response rate; CRR, Complete response rate; | | | | | | |
