# Supplementary material for: Sirolimus versus cyclosporine A in patients with primary acquired pure red cell aplasia: a prospective cohort study
Source: Blood Cancer J. 2023 May 10;13(1):74. doi: 10.1038/s41408-023-00845-3 (PMC10169841; doi:10.1038/s41408-023-00845-3)
Supplement: Supplementary file 3 — Table S2 [file 41408_2023_845_MOESM3_ESM.doc]

| **Table S2. Summary of adverse events** | | | | | | | |
| --- | --- | --- | --- | --- | --- | --- | --- |
| Complications (n, %) | All patients  (n = 56) | Sirolimus group (n = 26) | | Grades | CsA group (n = 30) | Grades | *P* |
| Infection | 6 (10.7%) | | 3 (11.5%) | 3 | 3 (10.0%) | 2 | 0.999 |
| Drug-related liver injury | 4 (7.1%) | | 1 (3.8%) | 2 | 3 (10.0%) | 2 | 0.991 |
| Drug-related renal injury | 7 (12.5%) | | 2 (7.7%) | 2 | 5 (16.7%) | 3 | 0.458 |
| Elevation of uric acid | 3 (5.4%) | | 1 (3.8%) | 2 | 2 (6.7%) | 2 | 0.809 |
| Gastrointestinal disorders | 5 (8.9%) | | 2 (7.7%) | 2 | 3 (10.0%) | 2 | 0.679 |
| Gingival hyperplasia | 3 (5.4%) | | 0 (0.0%) | - | 3 (10.0%) | 1 | 0.204 |
| Ulcer of the oral cavity | 3 (5.4%) | | 3 (11.5%) | 2 | 0 (0.0%) | 2 | 0.965 |
| Edema | 2 (3.6%) | | 1 (3.8%) | 1 | 1 (3.3% ) | 1 | 1.000 |
| Thrombosis | 2 (3.6%) | | 2 (7.7%) | 2 | 0 (0.0%) | - | 0.912 |
| Leukopenia | 2 (3.6%) | | 1 (3.8%) | 1 | 1 (3.3%) | 1 | 0.999 |
| Hyperglycaemia | 3 (5.4%) | | 2 (7.7%) | 1 | 1 (3.3%) | 1 | 0.809 |
