# Supplementary material for: Sirolimus versus cyclosporine A in patients with primary acquired pure red cell aplasia: a prospective cohort study
Source: Blood Cancer J. 2023 May 10;13(1):74. doi: 10.1038/s41408-023-00845-3 (PMC10169841; doi:10.1038/s41408-023-00845-3)
Supplement: Supplementary file 4 — Table S3 [file 41408_2023_845_MOESM4_ESM.doc]

| **Table S3. CD4^+^/CD8^+^T cell ratio, serum level of IL-6/8/10, TNF-α and EPO pre/post-treatment** | | | | | | |
| --- | --- | --- | --- | --- | --- | --- |
| Laboratory meters | Sirolimus group | | *P* | CsA group | | *P* |
|  | Baseline | 6-month Post |  | Baseline | 6-month Post |  |
| CD4^+^ / CD8^+^ T cell ratio | 0.60 ± 0.4 | 0.9 ± 0.2 | 0.31 | 0.8 ± 0.2 | 1.1 ± 0.1 | 0.26 |
| IL-6 (pg/ml) | 4.9 ± 0.8 | 4.2 ± 0.9 | 0.15 | 5.5 ± 1.1 | 4.1 ± 0.6 | 0.57 |
| IL-8 (pg/ml) | 16.7 ± 2.6 | 13.5 ± 3.0 | 0.43 | 14.6 ± 4.2 | 12.2 ± 4.6 | 0.67 |
| IL-10 (pg/ml) | 5.0 ± 0.0 | 5.6 ± 0.6 | 0.35 | 5.0 ± 0.0 | 5.3 ± 0.3 | 0.37 |
| TNF-α (pg/ml) | 11.7±1.1 | 7.5 ± 0.5 | 0.0073 | 13.4 ± 2.2 | 10.5 ± 2.9 | 0.17 |
| EPO (mIU/mL) | 516.2 ±104.4 | 229.9 ± 84.5 | 0.01 | 558.2 ± 107.3 | 629.5 ± 96.4 | 0.67 |
| IL-6/8/10, Interleukin 6/8/10; TNF-α, Tumor necrosis factors-α; EPO, Erythropoietin; | | | | | | |
